# Serum uric acid in pediatric metabolic assessment: the scientific rationale for routine screening and the urgent need for age- and sex-specific diagnostic thresholds

**DOI:** 10.3389/fneph.2026.1908272

**Published:** 2026-09-03

**Authors:** Izere Salomon, Yves NDAMUKUNDA, MUKANOHELI Solange, Eden Enyew Zeleke

**Affiliations:** 1 Department of General Medicine and Surgery, College of Medicine and Health Sciences, University of Rwanda, Kigali, Rwanda; 2 Department of Pediatrics, College of Medicine and Health Sciences, University of Rwanda, Kigali, Rwanda; 3 Department of Intensive Care Unit (ICU) Axon Neurology and Spine Center, Addis Ababa, Ethiopia; 4 Department of Intensive Care Unit (ICU) University of Gondar, Gondar, Ethiopia

**Keywords:** children, chronic kidney disease, hypertension, hyperuricemia, metabolic syndrome, paediatric screening, puberty, reference intervals

## Introduction

**Figure 1:**
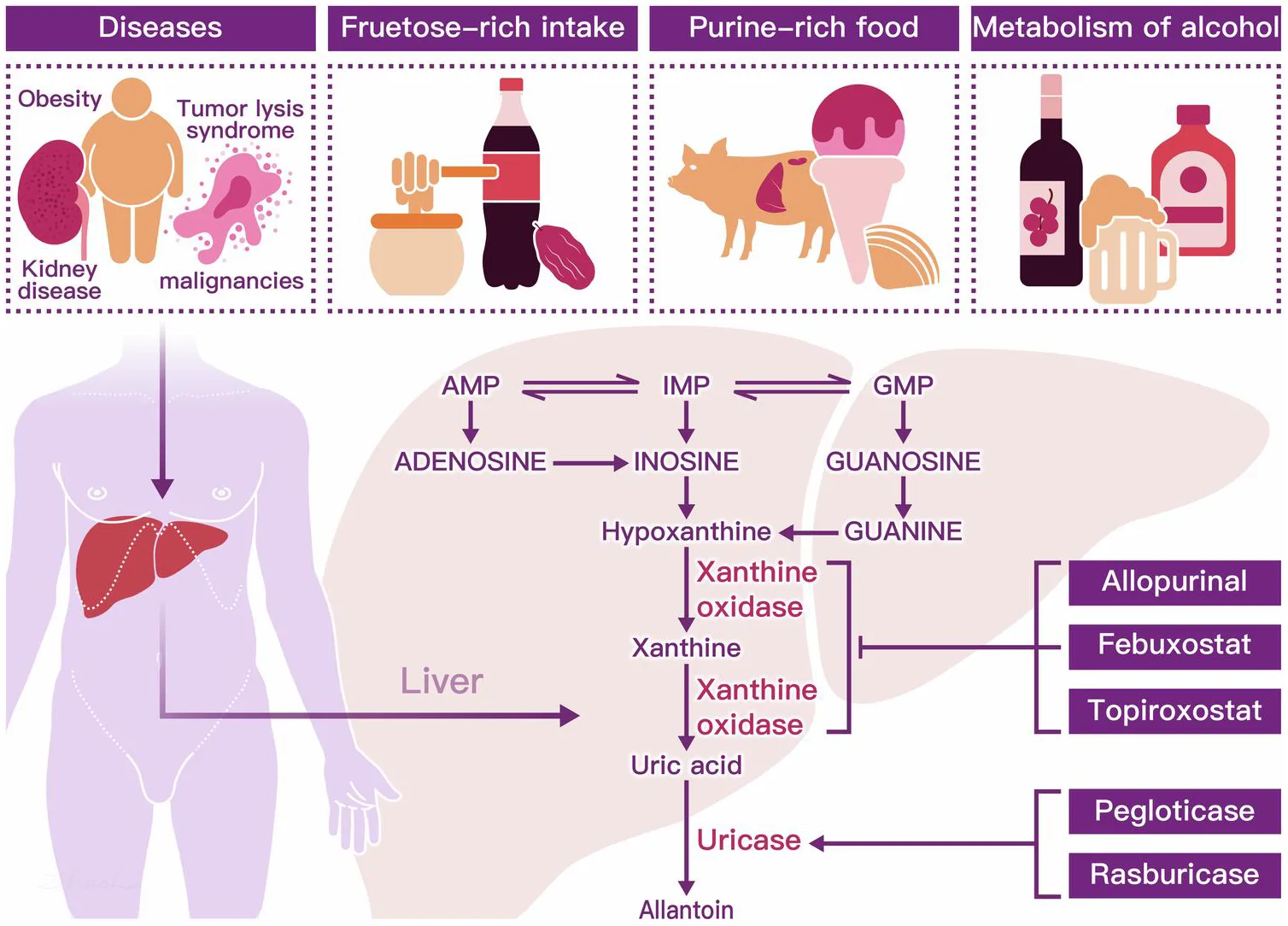
Overview of serum uric acid metabolism, determinants of hyperuricemia, and pharmacological targets for urate-lowering therapy (4). Serum uric acid is the end product of purine metabolism in humans. Purine nucleotides are degraded through hypoxanthine and xanthine to uric acid via xanthine oxidase within the liver. Hyperuricemia may result from increased purine turnover, fructose metabolism, purine-rich diets, alcohol consumption, obesity, malignancies, tumor lysis syndrome, or impaired renal excretion associated with chronic kidney disease. Xanthine oxidase inhibitors (allopurinol, febuxostat, and topiroxostat) reduce uric acid production, whereas recombinant uricase therapies (pegloticase and rasburicase) convert uric acid into the more soluble metabolite allantoin (31).

Serum uric acid (SUA) is the terminal product of purine catabolism in humans, generated through the sequential action of xanthine oxidoreductase on hypoxanthine and xanthine (**Figure 1**). Classically, elevation above accepted adult thresholds of 416 μmol/L (7.0 mg/dL) in males and 357 μmol/L(6.0 mg/dL) in females defines hyperuricemia and confers risk for gouty arthritis and nephrolithiasis. Over the past two decades, a growing body of epidemiological, pathophysiological, and interventional evidence has substantially broadened this picture. Elevated SUA is now consistently associated with hypertension, insulin resistance, metabolic syndrome, chronic kidney disease (CKD), and metabolic dysfunction-associated steatotic liver disease (MASLD) in both adults and children, though the nature and direction of these relationships vary across outcomes and, in several domains, causality remains an active area of scientific debate (1–3).

Serum uric acid homeostasis reflects the balance between purine metabolism, dietary and metabolic influences, and renal excretion. Hyperuricemia may result from increased uric acid production, reduced renal clearance, or both. In children, obesity, chronic kidney disease, fructose-rich diets, purine-rich foods, malignancies associated with increased cell turnover, and tumor lysis syndrome are among the principal contributors, as shown in **Figure 1**. These mechanisms converge on hepatic purine metabolism, where xanthine oxidase catalyzes the final steps leading to uric acid formation, providing the biological rationale for the use of xanthine oxidase inhibitors and uricase-based therapies in selected clinical settings.

Despite this evidence, SUA measurement remains largely absent from the routine pediatric metabolic panel in most international clinical guidelines. Its use in children is predominantly restricted to acute clinical contexts such as oncological tumor lysis syndrome and overt gouty arthritis endpoints that represent the extreme and late expression of what is often a prolonged subclinical process. Population data indicate that hyperuricemia affects a substantial and growing proportion of children globally, driven by the parallel epidemics of childhood obesity and increased dietary fructose consumption (5, 6). A pooled analysis of over 54,000 Chinese children and adolescents documented a hyperuricemia prevalence of 24.8% in 2016-2019, up from 16.7% in the preceding survey cycle (5), and prevalence estimates exceeding 50% have been reported in obese pediatric cohorts in some regions (6).

A critical obstacle to clinical action is the absence of validated, age- and sex-specific reference intervals for SUA in children. The adult diagnostic thresholds currently in use are not derived from pediatric populations, do not account for the progressive rise in SUA across childhood, and do not reflect the sex divergence that emerges only after puberty (7, 8). Applying fixed adult cut-offs to children of all ages and both sexes results in systematic misclassification, contributing in part to the clinical inertia that has surrounded this biomarker in pediatric practice.

The physiological basis of this age- and sex-dependent variation is well characterized and merits explicit statement before the epidemiological evidence is reviewed. SUA is relatively low in infancy, typically 2.2-2.5 mg/dL, reflecting a fractional excretion of uric acid (FE-UA) that exceeds 10% in the first months of life; FE-UA declines to approximately 8% by one year of age, after which SUA in most children stabilizes in the range of 3.5-4.5 mg/dL (9, 10). Renal tubular urate handling continues to mature throughout childhood, and after puberty, FE-UA falls further in boys but not in girls, resulting in the higher SUA concentrations characteristic of adolescent males relative to females (10). These maturational changes in renal urate handling, superimposed on the progressive rise in body weight and dietary purine intake across childhood, together explain why no single adult-derived threshold can be validly applied at any point across the pediatric age range, and why the age- and sex-stratified data reviewed below are indispensable rather than merely preferable.

This editorial argues that the cumulative evidence now supports incorporating SUA into routine pediatric metabolic screening in defined high-risk groups. In advancing this argument, we deliberately separate two distinct claims that are frequently conflated in the literature: SUA as a validated risk marker, for which the observational, mechanistic, and, in the case of hypertension, interventional evidence is now substantial, and SUA as a clinically actionable screening parameter with demonstrated downstream benefit, for which prospective screening-outcome trials in children do not yet exist. The recommendations that follow rest on the former claim; they should not be read as asserting the latter, and we return to this distinction explicitly before presenting the proposed framework. We review the epidemiological and interventional evidence linking elevated SUA to pediatric cardiometabolic and renal outcomes, examine existing data on age- and sex-specific reference intervals, consider the global applicability and practical implications of screening, and propose a minimum framework for clinical implementation.

## Epidemiology of hyperuricemia in children: a growing but under-quantified burden

Estimating the true prevalence of childhood hyperuricemia is complicated by the lack of a universally accepted pediatric definition, but available data consistently indicate that elevated SUA is common and rising. Rao et al. (2022), pooling data from 11 population-based Chinese studies comprising 54,580 participants aged 3–19 years, reported a prevalence that increased markedly with age from 3.7% at ages 3–5 years to 35.5% at ages 12–15 years and with weight status, reaching 64.5% among children with extreme obesity (5). A multicenter European clinical series of 13,890 children found hyperuricemia in 12.6% of the overall pediatric population, with obesity and CKD the most frequent associated conditions (9).

These figures must be interpreted in the context of considerable definitional heterogeneity. Across published studies, thresholds used to define pediatric hyperuricemia range from 5.5 mg/dL (327 μmol/L) to 7.0 mg/dL (416 μmol/L), with several studies using percentile-based cut-offs rather than fixed values. Dai et al. (2021), studying 5,439 healthy children aged 5–14 years in Southeast China using stringent exclusion criteria, demonstrated that the 97.5th percentile SUA in 14-year-old boys reaches 530.1 μmol/L, substantially above the adult male threshold of 416 μmol/L, while the corresponding value in girls is 439.0 μmol/L (7). Before age 10, SUA did not differ significantly between sexes, with 97.5th percentile values ranging from approximately 397-406 μmol/L in boys and 381-403 μmol/L in girls aged 5–9 years (7). These findings demonstrate that applying adult-derived thresholds to children across all age groups produces systematic misclassification in both directions, depending on age and sex.

Dietary fructose, independent of total caloric intake, represents a quantitatively important and modifiable driver of SUA elevation in children. Fructose undergoes rapid hepatic phosphorylation via fructokinase, transiently depleting adenosine triphosphate and accelerating purine catabolism, with urate as the terminal product (**Figure 1**). High-fructose corn syrup and sugar-sweetened beverages, whose consumption in children has increased substantially across multiple world regions, therefore promote hyperuricemia through a mechanism distinct from purine-rich dietary protein, partly explaining the burden of elevated SUA in children who are not overtly obese (11).

## Genetic and secondary causes of pediatric hyperuricemia: a necessary differential diagnosis

The associations reviewed in this editorial should not be taken to imply that an elevated SUA in a child is invariably a marker of cardiometabolic risk. Childhood hyperuricemia is aetiologically heterogeneous, and a purely metabolic interpretation risks overlooking rarer but clinically important causes. Persistent or early-onset hyperuricemia, particularly when accompanied by a family history of gout or nephrolithiasis at an unusually young age, should prompt consideration of inherited disorders of purine metabolism or renal urate handling, including uromodulin-associated kidney disease, glycogen storage disease type I, fructose-1,6-bisphosphatase deficiency, hereditary fructose intolerance, and mitochondrial disorders (12). Transient or medication-related hyperuricemia is more common still; thiazide diuretics, valproate, phenobarbital, and calcineurin inhibitors such as cyclosporine can each elevate SUA, as can acute conditions including gastroenteritis, dehydration, haemolytic anaemia, and malignancy-associated increased cell turnover. A structured pediatric screening pathway should therefore incorporate a basic differential diagnosis step rather than treating every elevated SUA result as evidence of an underlying cardiometabolic process.

Genetic determinants of urate transport further contribute to this heterogeneity and merit explicit consideration alongside metabolic and renal factors. Loss-of-function variants in ABCG2, which encodes the ATP-binding cassette transporter G2 responsible for extra-renal urate excretion, are among the best-characterized genetic contributors to hyperuricemia and gout across the age range. In a Czech cohort by Stiburkova et al. (2019) (13) analyzing pediatric-onset hyperuricemia and gout specifically, non-synonymous ABCG2 variants, principally the common p.Q141K (rs2231142) variant, were markedly overrepresented among children and adolescents with pediatric-onset disease; the minor allele frequency of p.Q141K was 38.7% in pediatric-onset patients compared with 21.2% in adult-onset patients (OR 2.4, p = 0.005) and 8.5% in normouricemic controls (OR 6.8, p < 0.0001), with *in vitro* functional studies confirming that the variant reduces ABCG2 transport capacity by approximately half (13). Incorporating ABCG2 status, or at minimum a family history suggestive of inherited urate transport dysfunction, into the evaluation of children with persistent or unexplained hyperuricemia would help distinguish those in whom elevated SUA reflects an inherited transport defect from those in whom it reflects an acquired cardiometabolic process, and the two are not mutually exclusive.

## Serum uric acid and cardiometabolic risk in children: strengths and limitations of the evidence

The association between elevated SUA and metabolic syndrome in children is one of the most consistently replicated observations in pediatric metabolic research. Ford et al. (2007), analyzing data from 1,370 US adolescents aged 12–17 years in the NHANES 1999–2002 survey, reported that the prevalence of metabolic syndrome rose from below 1% in the lowest SUA quartile to 21.1% in the highest (OR 14.79; 95% CI 7.78-28.11), remaining significant after adjustment for age, sex, race, and C-reactive protein (14). A meta-analysis of 34 pediatric studies confirmed pooled correlations between SUA and fasting blood glucose (r = 0.24), fasting insulin (r = 0.26), triglycerides (r = 0.23), and an inverse correlation with high-density lipoprotein cholesterol (HDL-C) (r = -0.28) (15). These are robust associations across diverse populations, though the cross-sectional design of most contributing studies limits conclusions about temporal sequence and directionality.

These associations are also consistently observed in Latin American pediatric populations, underscoring the global relevance of hyperuricemia as a cardiometabolic risk marker. In a Mexican cohort of 59 obese prepubertal children, higher SUA concentrations were positively associated with insulin resistance and hypertriglyceridemia and inversely associated with HDL-C. Moreover, each 1 mg/dL increase in SUA was associated with an approximately threefold higher likelihood of meeting the diagnostic criteria for metabolic syndrome (MetS) (16). Similarly, a large Brazilian cohort involving 1,750 children and adolescents demonstrated that serum uric acid concentrations at or above the 90th percentile were independently associated with substantially increased odds of overweight and obesity (OR 3.6) (16, 17). More recently, Lorra et al. (2026) (18) further reinforced the role of SUA as an early metabolic marker among adolescents by examining 4,390 Brazilian participants aged 12–17 years. Higher SUA quartiles were consistently associated with an adverse cardiometabolic profile, including greater waist circumference, higher BMI Z-scores, elevated blood pressure, increased triglyceride levels, and higher total and low-density lipoprotein concentrations. Together, these findings indicate that the cardiometabolic implications of pediatric hyperuricemia extend beyond Asian, North American, and European populations, highlighting its relevance across diverse ethnic and geographic contexts experiencing rapid nutritional and epidemiological transitions.

For cardiovascular outcomes, particularly hypertension, the evidence is comparatively stronger and includes longitudinal and interventional data. The Bogalusa Heart Study demonstrated that childhood SUA independently predicted adult blood pressure after adjustment for body mass index, age, sex, and race, with SUA trajectory over time serving as an additional predictor of adult systolic pressure (19). The Ewha Birth and Growth Cohort Study further showed that children with SUA above the median at age three had significantly higher blood pressure at age seven, with those maintaining elevated SUA at both three and five years carrying the greatest blood pressure burden at follow-up (20). Mechanistically, uric acid activates the renin-angiotensin-aldosterone system (RAAS), increases renal sodium reabsorption, and inhibits endothelial nitric oxide synthesis, collectively promoting vasoconstriction through pathways that may become self-sustaining once established (21).

Critically, the interventional literature provides the most direct evidence linking SUA reduction to a measurable pediatric cardiovascular outcome. In a randomized, double-blind, placebo-controlled crossover trial, Feig et al.(2008) enrolled 30 adolescents aged 11–17 years with newly diagnosed stage 1 essential hypertension and SUA >/= 6 mg/dL, and demonstrated that allopurinol 200 mg twice daily reduced mean systolic blood pressure by 6.9 mmHg versus 2.0 mmHg with placebo (p = 0.009), with blood pressure normalizing in 20 of 30 adolescents during active treatment compared with 1 during placebo (22). A subsequent trial in 60 obese pre-hypertensive adolescents confirmed that urate-lowering therapy reduced systolic blood pressure by approximately 10 mmHg, significantly exceeding the placebo response (23). These trials support a causal role for uric acid in blood pressure elevation in adolescents, a conclusion that is further strengthened by the biological plausibility of the proposed mechanism. For CKD progression and MASLD, however, the evidence base remains predominantly observational, and causality should be characterized as biologically plausible and epidemiologically supported rather than established.

Subclinical vascular injury may precede clinical hypertension. Genovesi et al. (2022) found that SUA was independently associated with increased arterial stiffness, measured by pulse wave velocity, in children and adolescents, with the relationship mediated in part through insulin resistance and blood pressure (21). These data collectively support SUA as a clinically relevant cardiometabolic marker in children, with the cardiovascular domain providing the strongest and most actionable evidence for clinical attention.

## Serum uric acid and renal outcomes in children: prognostic signal and causal uncertainty

The kidney is simultaneously a principal regulator of urate excretion and a proposed target of urate-mediated injury. Experimental models demonstrate that mild urate elevation induces afferent arteriolar constriction, glomerular hypertrophy, tubular fibrosis, and renin-angiotensin axis activation in renal tissue through crystal-independent mechanisms, changes that are reversed by xanthine oxidase inhibition in animal studies (24). Whether these mechanisms translate directly to CKD progression in humans and in children specifically remains an important and unresolved question.

The most substantive clinical evidence comes from the CKD in Children (CKiD) cohort study, which enrolled 678 children with established CKD across 52 North American sites. Hyperuricemia was independently associated with a composite renal endpoint of either a greater than 30% decline in estimated glomerular filtration rate or initiation of renal replacement therapy, after multivariable adjustment for blood pressure, proteinuria, CKD etiology, and body mass index, with the steepest decline seen in children with SUA above 7.5 mg/dL (2). These are important prognostic associations, but the observational design precludes causal inference. No adequately powered randomized trial has yet tested whether urate-lowering therapy slows CKD progression in children, and this remains a critical evidence gap.

A Korean pediatric CKD cohort further identified a U-shaped relationship between SUA and hypertension and left ventricular hypertrophy prevalence, with the lowest risk at SUA levels between 5.5 and 7.5 mg/dL (25). This pattern suggests that both extremes of SUA are associated with adverse outcomes in children with CKD, and argues for targeted monitoring within a defined range rather than simple threshold-based intervention. In practical terms, it also means that SUA adds independent prognostic information beyond creatinine, eGFR, and proteinuria in pediatric CKD, a finding that supports its routine measurement in this population even in the absence of established causal evidence.

Among children with hematological malignancies, Down syndrome, and congenital heart disease, SUA elevations are more prevalent and the renal consequences potentially more significant, yet longitudinal SUA monitoring is not part of standardized follow-up protocols in most of these groups (9). This represents a tractable gap in current pediatric nephrology practice that does not depend on resolving causal questions before being addressed.

## The reference interval problem: why current thresholds misclassify children

The most frequently cited obstacle to incorporating SUA into routine pediatric metabolic screening is the absence of validated, age- and sex-specific diagnostic thresholds for children. The adult cut-offs of 416 μmol/L for males and 357 μmol/L for females are anchored in adult population data and reflect the physicochemical urate solubility threshold. They are not derived from children, do not account for the developmental trajectory of SUA across childhood, and do not reflect the sex divergence that emerges only with the hormonal shifts of puberty rather than at a fixed chronological age.

Dai et al. (2021) provide some of the most detailed age- and sex-stratified SUA reference data currently available (7). Studying 5,439 healthy children aged 5–14 years in Southeast China with stringent exclusion criteria, their data yielded three clinically instructive findings. First, SUA increases progressively with age in both sexes throughout childhood. Second, no significant sex difference exists before age 10; divergence emerges from ages 10–11 onward, with boys’ SUA rising substantially faster, consistent with the uricosuric effects of estrogen and the urate-retaining effects of testosterone. Third, the 97.5th percentile SUA in healthy pre-pubertal children is considerably below adult thresholds; approximately 397 μmol/L in 5-year-old boys and 381 μmol/L in 5-year-old girls (7).

Multiple linear regression in the Dai et al. cohort yielded sex-specific SUA prediction equations incorporating both BMI and age: in boys, mean SUA (μmol/L) = 109.321 + (BMI x 7.69) + (age in years x 7.76); in girls, mean SUA=177.848 + (BMI x 5.623) + (age x 2.407) (7). The stronger BMI coefficient in boys and the stronger age coefficient in girls formally quantify the differential contributions of adiposity and hormonal maturation to urate production across the two sexes. Complementary reference interval data from the United States, Australia, and additional Chinese cohorts show that the 97.5th percentile SUA in healthy post-pubertal boys approaches or exceeds 500 μmol/L across diverse populations, substantially above the 416 μmol/L adult threshold, confirming that applying fixed adult cut-offs to adolescent males will systematically underdiagnose clinically significant elevations in this group (7, 8).

Independent corroboration comes from the Canadian Laboratory Initiative on Pediatric Reference Intervals (CALIPER) (26)The most rigorously constructed pediatric reference interval database currently in existence was established through more than a decade of prospective data collection from over 12,000 healthy children across Canada, using standardized analytical platforms. The CALIPER white paper by Adeli et al. (2017) explicitly identified SUA as one of the analytes exhibiting the most pronounced age-related trends, with concentrations substantially lower in the pediatric compared to the adult population, a finding the authors highlighted as a direct patient safety concern, noting that the use of inappropriate adult reference intervals carries a measurable risk of misclassifying children as normal when their results are, in fact, elevated relative to their age-appropriate peers, or vice versa (26). CALIPER-derived SUA reference intervals used in clinical practice further confirm the magnitude of this divergence from adult norms; for children aged 1 to under 12 years, the reference range is 1.7-4.7 mg/dL in both sexes, rising to 2.5-7.5 mg/dL in males and 2.5-5.7 mg/dL in females aged 12 to under 19 years (26). That these Western pediatric data converge closely with the Chinese cohort findings of Dai et al. across entirely independent populations, analytical platforms, and methodological frameworks substantially strengthens the case that the age- and sex-related trajectory of SUA in childhood is a biological constant rather than a population-specific artefact, and that its clinical implications are globally generalizable.

The field urgently requires internationally harmonized reference intervals stratified by age, sex, and pubertal stage, derived from diverse ethnic and geographical populations, and validated against clinical outcomes rather than statistical distribution alone. The CALIPER programme offers a replicable methodological model for how this can be achieved, including prospective community recruitment, standardized pre-analytical conditions, multi-platform validation, and open-access dissemination through a queryable database and mobile reference application. Extending this model to include LMIC populations and to validate SUA thresholds against longitudinal cardiovascular and renal outcomes, rather than against statistical distribution alone, represents the most important single methodological step toward making pediatric SUA screening clinically actionable at a global scale (26).

## Global applicability: screening in low- and middle-income settings

Most of the evidence reviewed in this editorial originates from China, the United States, Europe, and, to a lesser extent, the Middle East and South Korea. This geographical concentration reflects the distribution of published pediatric metabolic research globally and should not be interpreted as evidence that childhood hyperuricemia is confined to high-income settings. On the contrary, the drivers of hyperuricemia in children, childhood obesity, increased fructose consumption, sedentary behavior, and early-onset metabolic syndrome are documented as rising rapidly across sub-Saharan Africa, South Asia, and Latin America, regions where the epidemiological transition in childhood nutrition is well advanced (27, 28).

In low- and middle-income countries (LMICs), the case for SUA screening carries both additional justification and additional complexity. The potential benefit is substantial; in settings where early-stage CKD and hypertension in children are often missed until advanced disease, an inexpensive biomarker that flags cardiometabolic and renal risk earlier than conventional markers could have meaningful public health value. The SUA assay is technically straightforward, requires no specialized equipment beyond standard clinical biochemistry, and is already performed routinely in most district and tertiary hospitals across Africa and South Asia for adult gout and oncology applications.

The complexity lies in interpretation. Reference interval data from East Asian and European children may not be directly transferable to sub-Saharan African or South Asian pediatric populations, in whom differences in body composition, pubertal timing, dietary purine intake, and renal physiology are plausible. No published pediatric SUA reference interval study from sub-Saharan Africa was identified in preparing this editorial, a gap that itself requires correction before LMIC-specific screening thresholds can be proposed with confidence. In the interim, clinicians in resource-limited settings should apply age- and sex-stratified reference data from the most contextually comparable available populations while recognizing this limitation explicitly.

The call for internationally harmonized pediatric SUA reference intervals articulated in this editorial is therefore not only a scientific priority but a matter of global equity. Research initiatives targeting LMIC pediatric populations, including those embedded within existing child health cohort studies in Africa, South Asia, and Latin America, should be supported and funded with this gap in view.

## A proposed framework for serum uric acid screening in pediatric practice: justification, limitations, and cost considerations

SUA measurement is performed by a standard colorimetric enzymatic assay available in virtually every clinical biochemistry laboratory globally, requiring no additional specimen or infrastructure beyond a standard fasting blood draw. In settings where children already undergo fasting metabolic evaluation for obesity, hypertension, CKD monitoring, or MASLD assessment, the marginal cost of including SUA is negligible. The SUA assay is among the least expensive tests in the standard clinical biochemistry repertoire, routinely priced at a fraction of other first-line metabolic investigations; no dedicated equipment, extended turnaround time, or specialist laboratory is required. A formal cost-effectiveness analysis specifically evaluating SUA screening in children has not yet been published and represents a gap in the health economics literature that should be addressed alongside the clinical evidence base. Given that a single SUA result provides independent prognostic information across cardiovascular, renal, and hepatic risk domains simultaneously, the informational yield per unit cost compares favorably with other candidate additions to the routine pediatric metabolic screen.

Consistent with the distinction drawn in the introduction between SUA as a risk marker and SUA as a validated screening parameter, before presenting a screening framework it is important to state explicitly what the current evidence does and does not support. The evidence reviewed in this editorial demonstrates that elevated SUA in children is associated with cardiometabolic and renal risk, that it carries independent prognostic information in defined high-risk populations, and that urate-lowering reduces blood pressure in hyperuricemic adolescents with hypertension. It does not yet demonstrate, through prospective screening trials, that identifying and treating hyperuricemia in asymptomatic children improves long-term cardiovascular or renal outcomes at a population level. This distinction matters; the recommendation to screen is based on prognostic and mechanistic evidence, not on completed screening outcome trials, and this limitation should be communicated transparently to clinicians and policymakers.

Potential harms of screening also deserve acknowledgement. Identification of an elevated SUA in an asymptomatic child carries risks of parental anxiety, unnecessary specialist referral, and, most importantly, premature or inappropriate pharmacological treatment in the absence of clinical indication. The evidence reviewed does not support urate-lowering pharmacotherapy in asymptomatic children with isolated hyperuricemia outside of specific high-risk contexts such as progressive CKD or uncontrolled hypertension. The 2025 Consensus Statement on Adolescent Hyperuricemia and Gout explicitly cautions against routine pharmacological treatment of asymptomatic hyperuricemia in adolescents based on current evidence (29). A well-designed screening programme would therefore require clear clinical decision pathways specifying when an elevated result warrants lifestyle intervention alone, when specialist review is appropriate, and when pharmacotherapy should be considered, pathways that currently exist only in partial and non-standardized form.

Based on the evidence reviewed, with these caveats acknowledged, we propose the following minimum clinical framework:

- SUA measurement should be included in the routine fasting metabolic panel of all children with overweight or obesity (BMI >/= 85th centile for age and sex), at initial assessment and at annual metabolic review, given the consistent and strong association between elevated SUA and metabolic syndrome components in this group and the low cost of measurement.
- SUA should form part of the standard baseline evaluation of any child presenting with new-onset hypertension, regardless of obesity status, where the interventional trial data provide the strongest available justification for measurement and potential treatment.
- SUA should be measured routinely alongside serum creatinine, eGFR, and urinary protein in children with established CKD of any etiology, where the CKiD cohort data support its use as an independent prognostic biomarker (2), while recognizing that the case for urate-lowering therapy to slow CKD progression awaits pediatric interventional trial evidence.
- In children with suspected or confirmed MASLD, SUA should be measured as part of metabolic characterization, acknowledging that the MASLD association is biologically plausible and epidemiologically supported but not yet established as causal in pediatric populations.
- Results should be interpreted using age- and sex-specific reference data rather than adult thresholds. Until internationally validated pediatric reference intervals are available, the published age- and sex-stratified data of Dai et al. (7) and comparable cohort studies provide the most appropriate available reference framework for clinical interpretation.

When elevated SUA is identified, the initial response should prioritize lifestyle modification, specifically reduction of fructose-rich foods and sugar-sweetened beverages, and promotion of regular physical activity, with pharmacological urate-lowering therapy reserved for children with specific clinical indications, and only after discussion of the limited pediatric evidence base with families (29, 30).

## Conclusion

Serum uric acid is an inexpensive, biochemically accessible marker whose consistent associations with hypertension, insulin resistance, metabolic syndrome, CKD, and MASLD in children are supported by a substantial and geographically diverse body of evidence. For cardiovascular outcomes and hypertension specifically, interventional data demonstrating blood pressure reduction following urate-lowering therapy in adolescents support a causal interpretation. For CKD progression and MASLD, the evidence remains predominantly observational, and causality should be considered biologically plausible rather than proven. The primary barriers to clinical implementation are the absence of harmonized, age- and sex-specific diagnostic thresholds for children, the lack of LMIC-derived reference data, and the absence of screening outcome trials demonstrating that identifying hyperuricemia in asymptomatic children improves long-term health.

These are resolvable gaps. The data needed to construct pediatric-specific reference intervals across diverse populations already exist in nascent form and require coordinated international synthesis. Screening outcome trials in high-risk pediatric groups are feasible and warranted. Pharmacological trials in pediatric CKD and obesity-related hypertension remain the most urgent therapeutic research priority. In the interim, clinicians should measure SUA in children at cardiometabolic and renal risk, interpret results against age- and sex-appropriate reference data, and resist both the uncritical application of adult thresholds and the premature initiation of pharmacotherapy in asymptomatic patients. A biomarker of this accessibility, informational breadth, and low cost deserves a defined and evidence-guided role in pediatric metabolic care, and the evidence now available is sufficient to begin building that role.
